# Effect of Different Types of Aluminosilicates on the Thermo-Mechanical Properties of Metakaolinite-Based Geopolymer Composites

**DOI:** 10.3390/polym14224838

**Published:** 2022-11-10

**Authors:** Jan Kohout, Petr Koutník, Pavlína Hájková, Eliška Kohoutová, Aleš Soukup

**Affiliations:** 1 ORLEN UniCRE a.s., Revoluční 1521/84, 400 01 Ústí nad Labem, Czech Republic; 2 Department of Material Science, Faculty of Mechanical Engineering, Technical University of Liberec, Studentská 1402/2, 461 17 Liberec, Czech Republic

**Keywords:** metakaolinite, geopolymer, metakaolin, claystone, characterization, thermal properties, mechanical properties

## Abstract

In this study, the effect of different types of aluminosilicates on the thermo-mechanical properties of metakaolinite-based geopolymer binders and composites was examined. The metakaolinite-based geopolymer binders and composites were produced from three different types of aluminosilicates (one metakaolin and two calcined claystones) and a potassium alkaline activator. Chamotte was added as a filler, amounting to 65% by volume, to create geopolymer composites. Geopolymer binders were characterized by X-ray diffraction, rotary rheometer and scanning electron microscopy. The mechanical properties, thermal dilatation and thermal conductivity were investigated on geopolymer composites with three different aluminosilicates before and after exposure to high temperatures (up to 1200 °C). The results showed that the geopolymer binders prepared from calcined claystones had a lower dynamic viscosity (787 and 588 mPa·s) compared to the geopolymer binders prepared from metakaolin (1090 mPa·s). Geopolymer composites based on metakaolin had lower shrinkage (0.6%) and higher refractoriness (1520 °C) than geopolymers from calcined claystones (0.9% and 1.5%, 1500 °C and 1470 °C). Geopolymers based on calcined kaolinitic claystones are a promising material with higher compressive (95.2 and 71.5 MPa) and flexural strength (12.4 and 10.7 MPa) compared to geopolymers based on metakaolin (compressive strength 57.7 MPa).

## 1. Introduction

Geopolymers, as inorganic materials, are based on the alkaline activation of aluminosilicates [1,2]. Geopolymers have attracted attention as a sustainable alternative material whose production is associated with low CO_2_ emissions and low energy consumption compared to materials based on Portland cement and ceramics [3,4]. Therefore, geopolymers have been extensively studied in recent years [5,6,7]. Geopolymers possess excellent mechanical properties, durability and resistance to high temperatures and chemicals (acids and organic solvents) [8,9,10]. A wide variety of geopolymer applications have been developed in industrial practices, such as building materials [11], decorative and restoration materials [12], immobilizers of toxic waste [13], materials for 3D printing [14,15], catalysts [16], coatings [17] and fiber-reinforced geopolymer composites [18,19,20].

Geopolymers are formed by the partial dissolution of powdered aluminosilicates in a liquid alkaline activator and the subsequent polycondensation reaction of hydrolyzed silicates and aluminates into a three-dimensional polymer network, which leads to the hardening of the binder [21,22,23]. The most common aluminosilicate materials for geopolymerization are fly, rice husk or volcanic ash, blast furnace slag, metakaolin or demolition wastes [1,24,25,26,27,28]. An aqueous alkali metal hydroxide or liquid alkali silicate (water glass) is usually used as an alkaline activator [29].

The type of aluminosilicate [7,30,31], type of alkali cation (Na^+^ or K^+^) [32], Si/Al molar ratio [33,34], Si/Na or Si/K molar ratio [35,36], water content [11,25,37] and the curing conditions [38,39] have a significant influence on the properties of geopolymers. The ability of aluminosilicates to dissolve in an alkaline activator has a significant effect on the process of geopolymerization and the resulting properties of the geopolymer. The undissolved residue directly affects the physical properties of the geopolymer because it is incorporated as part of the resulting geopolymer [1,26,40,41]. The most popular and most often studied raw aluminosilicates for the preparation of geopolymers are fly ash and metakaolin. Fly ashes are cheap and available worldwide in sufficient quantities as industrial by-products, but their chemical composition and physical properties are very variable. In contrast, commercial metakaolin is more expensive and manufactured by controlled calcination of natural kaolin with a high content of kaolinite. Consistent chemical composition and properties are the main advantages of metakaolin [1,31,42].

Despite the fact that metakaolins generally have similar chemical composition, there have only been a few reported studies aimed at the comparative performance of geopolymers prepared from different metakaolins. The relationship between the characteristics of metakaolin samples and the properties of geopolymers have been studied by Kuenzel et al. [31]. No dependence was found between the aluminum content in metakaolin samples and geopolymer setting time, heat output or mechanical strength. San Nicolas et al. [43] compared two different preparation methods (flash and standard calcination) of three metakaolins. They discovered that flash calcination affected the physical properties but did not change the chemical composition of metakaolins compared to standard calcination. Xu and Van Deventer [44] investigated the behavior of various aluminosilicate minerals in geopolymerization. They found out that the molar ratio of Si:Al in the raw material had a significant effect on the compressive strength. Rovnaník et al. [7] compared the thermal behavior and mechanical properties of geopolymers prepared from metakaolin or fly ash. They reported that geopolymer composites prepared from metakaolin showed higher values of mechanical properties compared to fly ash geopolymers when they were tested under laboratory conditions. Most of the works have, however, focused only on metakaolin as the source of aluminosilicate and an investigation of mechanical properties and chemical/phased composition. Very few studies have been reported comparing metakaolin with different types of aluminosilicates. Almost no research has investigated the effect of aluminosilicates on the behavior of geopolymers at high temperatures, let alone testing the compressive strength in situ.

The motivation for the present work is to compare the effect of different types of raw Al and Si rich materials on the thermo-mechanical properties of geopolymer binders and composites based on metakaolinite (filled with chamotte aggregate as the most suitable option for this study). Geopolymer binders were prepared from calcined kaolin or kaolinitic claystones and a potassium alkaline activator.

## 2. Materials and Methods

### 2.1. Materials

**Figure 1:**
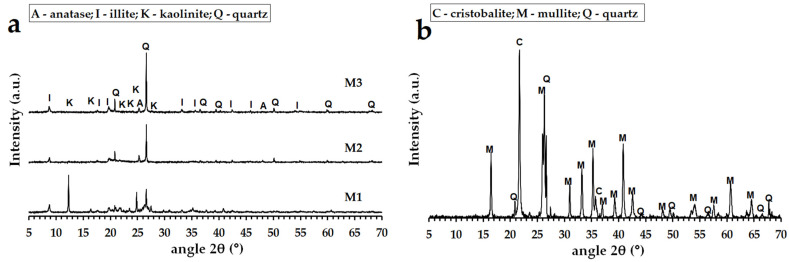
XRD patterns of the aluminosilicate raw materials (**a**) and chamotte (**b**).

**Figure 2:**
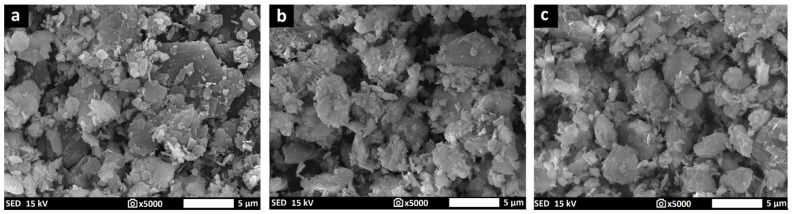
The morphology of M1 (**a**), M2 (**b**) and M3 (**c**).

Geopolymer binders were synthesized from commercial metakaolin Mefisto K_05_ and two metakaolinite-rich materials, Mefisto L_05_ and Mefisto LB_05_, produced by the calcination of kaolin or kaolinitic claystone at about 750 °C in a rotary furnace (České lupkové závody, a.s., Nové Strašecí, Czech Republic). The samples of the materials were named in the above-mentioned order as M1 to M3. The alkaline activator was prepared from potassium silicate (specific gravity 1384 kg/m^3^, Vodní sklo, a.s., Prague, Czech Republic) and potassium hydroxide pellets (G.R. grade, 88.2 wt % KOH, Lach-Ner, s.r.o., Neratovice, Czech Republic). The heat-resistant filler used to prepare geopolymer composites was chamotte (České lupkové závody, a.s., Nové Strašecí, Czech Republic) of particle size 0–2 mm. Table 1 shows the chemical compositions of the raw materials used. Table 2 displays the physical properties of the raw materials used. Figure 1 presents the results of X-ray diffraction analysis (XRD, Bruker, Billerica, MA, USA) of aluminosilicate raw materials and chamotte as an aggregate. Images of the morphology of aluminosilicate raw materials examined by a scanning electron microscope (SEM, JEOL, Tokyo, Japan) are given in Figure 2.

### 2.2. Preparation of Geopolymer Samples

The alkali activator was obtained by mixing solid potassium hydroxide with a potassium silicate solution. The aluminosilicate materials were dried at 110 °C for 24 h in order to remove the water absorbed during the milling and storage. Geopolymer binders were obtained by mixing the aluminosilicate raw material with an alkali activator and additional distilled water in a planetary mixer at laboratory temperature for 10 min in order to prepare a homogenous mixture. The weight ratio of the aluminosilicate component to the alkali activator was 40:60 for all the raw aluminosilicate materials. The homogenous slurry was poured into silicon molds and vibrated for 5 min in order to remove air bubbles. The prepared samples were cured at 60 °C for 4 h in an electric oven in sealed polyethylene bags. The samples were de-molded and left to harden at laboratory temperature (LT, 20 °C) for 7 days. The curing conditions, including time, were selected on the basis of the work of Rovnaník et al. [38], which verified that, under these conditions, the samples reached optimal strengths.

The obtained geopolymer binders had a molar ratio of K:Al 1 and total water content of 30%. The Si:Al molar ratio of geopolymer binders was 1.6 (M1), 1.5 (M2) and 1.7 (M3). The different Si:Al molar ratios were given by the different composition of SiO_2_ and Al_2_O_3_ in the raw aluminosilicate materials. The raw aluminosilicate materials and geopolymer binders were selected on the basis of the results of our previous studies. The geopolymer binders with Mefisto L_05_ as a source of aluminum and silicon provided binders with very low viscosity and excellent mechanical properties [25,45]. The geopolymer binders were named as GB-X, where X indicates the aluminosilicate materials used.

The chamotte aggregate was mixed into the geopolymer binder and mixed for an additional 5 min to form the geopolymer composites. The amount of chamotte aggregate added was 65% by volume. The curing conditions were the same as for geopolymer binders. The addition of the aggregate was chosen based on our results from previous work. Geopolymer composites filled with chamotte provide composites with low shrinkage, high heat resistance and excellent mechanical properties [46]. The composites with chamotte were named as GS-X, where X indicates the aluminosilicate materials used.

The samples of geopolymer composites were heated in an electric furnace (Clasic, type 5013V, Řevnice, Czech Republic) to temperatures of 200, 400, 600, 800, 1000 and 1200 °C at a constant heating rate of 5 °C/min after hardening for 7 days. The samples were kept at the given temperature for 1 h. The samples were kept inside the furnace until they reached laboratory temperature. The samples were named as GS-X-Y, where Y indicates the temperature exposure in °C.

### 2.3. Analytical and Testing Methods

X-ray fluorescence (XRF, Bruker, Billerica, MA, USA) with a BRUKER S8 Tiger instrument was used to identify the chemical compositions of the solid raw materials. X-ray patterns were collected from the powdered raw materials and tested samples after milling, and the patterns were obtained from 5° to 70° (2θ) applying a BRUKER D8 Advanced X-ray diffraction system provided by a BRUKER SSD 160 detector working with Cu-Kα radiation with an X-ray source at 40 kV and 25 mA. The XRD patterns were obtained using a dwelling time of 1 s and a step size of 0.02° (2θ).

Specific gravity was determined by a gas (He) pycnometer Pycnomatic ATC Evo (Microtrac, Osaka, Japan).

An Autosorb iQ (Quantochrome Instruments, Boynton Beach, FL, USA) was used to measure the Brunauer–Emmett–Teller (BET) surface area of the aluminosilicate materials by nitrogen adsorption.

The content of elements and the K/Na ratio in the liquid potassium silicate were determined by an inductively coupled plasma optical emission spectrometer (ICP-OES) OPTI-MA 8000 (Perkin Elmer, Waltham, MA, USA). Conventional acid-base titration methods were used to identify the total content of alkali metals (Na, K) and SiO_2_ in potassium silicate solutions. These methods were selected due to their better accuracy at higher concentrations.

Mastersizer 3000 laser diffraction particle size analyzer (MALVERN Instruments, Malvern, UK) was used to determine particle size distribution of the raw aluminosilicate materials. Agglomerates were broken by ultrasound treatment.

Rotary rheometer Rheotest RN 4.1 (Rheotest Medingen, Ottendorf-Okrilla, Germany) was used in order to determine the dynamic viscosities of the geopolymer binders at 25 °C using a 38 mm diameter cylinder at a shear rate of 300 s-1 for 300 s. SEM JSM-IT500HR from JEOL (JEOL, Tokyo, Japan) was used in order to display the morphology of the raw aluminosilicate materials and geopolymer binders. The samples were coated with a thin layer of gold (5 nm) to make them conductive. Representative secondary electron images were taken in high vacuum mode using an accelerating voltage of 15 kV and under magnification of 2500× (scale 10 µm) for the prepared samples and 5000× (scale 5 µm) for the raw materials used.

Automatic apparatus Vicatronic from MATEST (MATEST, Treviolo, Italy) was used to determine the initial, final and real setting times. The measurement was carried out according to standard EN 480–2. The apparatus was kept during the test in a climatic chamber at 95 ± 5% relative humidity and 25 ± 5 °C and at 60 °C. Dilatometric characterization was performed with a dilatometer (Clasic CZ, type DIL 1500, Řevnice, Czech Republic) up to 1200 °C in static air (heating rate 5 °C/min) on 20 × 20 × 160 mm samples of geopolymer composites.

A heating microscope (Clasic CZ, type 0116 VAK, Řevnice, Czech Republic) was used for determination of the pyrometric cone refractoriness of geopolymer composites. The measurement was carried out according to European standard EN 993-12. The dimensions of the cone samples were 30 × 8.5 mm. The temperature was growing at a rate of 5 °C/min. The tested cone was placed between two reference cones with different melting points, and melting was observed. Isomet 2144 (APPLIED PRECISION, Bratislava, Slovakia) was used in order to determine the thermal conductivity of geopolymer composites.

Mechanical properties were tested on a universal testing machine, LabTest 6.200 (Labortech, Opava, Czech Republic). An electric furnace, allowing the testing of mechanical properties in situ at temperatures up to 1200 °C, was part of the testing machine. A three-point bending test was used to investigate flexural strength. Six samples (20 × 20 × 160 mm) of geopolymer composites were tested before and after exposure to high temperatures up to 1200 °C with a crosshead speed of 0.1 MPa/s. The measurement of compressive strength and modulus of elasticity was carried out according to the ISO 1920-10 standard. Six samples (30 × 30 × 64 mm) of geopolymer composites were examined before and after exposure to high temperatures up to 1200 °C with a crosshead speed of 0.5 MPa/s. The measurements of compressive strength and modulus of elasticity in situ of the geopolymer composites at temperatures from 25 to 1200 °C were also taken. The temperature was growing at a rate of 5 °C/min and lasted 1 h at each tested temperature. Mechanical properties were measured 7 days after preparation.

## 3. Results and Discussion

### 3.1. Geopolymer Binders’ Characteristics

**Figure 3:**
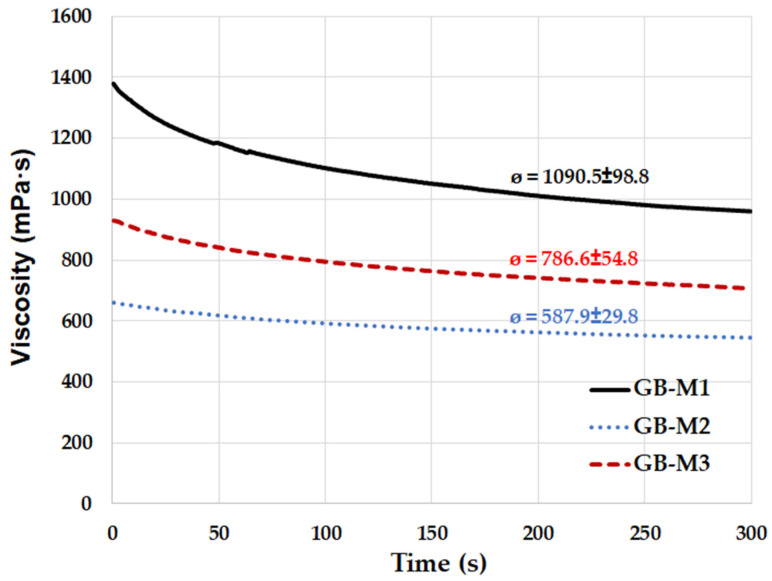
Viscosity measurement of geopolymer binders with different type of aluminosilicate.

The influence of the aluminosilicate on the dynamic viscosity of the geopolymer binder was examined for all metakaolinite raw materials, as shown in Figure 3. Geopolymer binders prepared from raw materials of M2 and M3 (calcined kaolinitic claystones) had significantly lower dynamic viscosity compared to the geopolymer binder prepared from M1 (metakaolin). The difference in the morphology of the particles shown in Figure 2 is probably the reason for the variable viscosity. Sample M1 contained particles with a higher aspect ratio (the ratio of the largest dimension of the particle to the smallest dimension) compared to particles of samples prepared from kaolinitic claystones. The reason is the higher arrangement of kaolinite plates in kaolin, which break up into individual plates more easily during grinding. All results of viscosity that were measured confirmed the expected successive decline in viscosity of geopolymer binders based on metakaolinite. Successive decline has already been reported and affiliated with partial dissolution of metakaolinite particles in the alkaline activator [47].

The results of initial, final and real setting time of geopolymer binders measured at two different temperatures are shown in Table 3. The temperature at setting time had an effect on the hardening of geopolymer binders; the initial setting time was reduced by up to 8× and real setting time by up to 12× at an elevated temperature. The rate of chemical reactions strongly depends on temperature, and therefore, geopolymer binders harden faster at higher temperatures. The results of setting time at a higher temperature match the results found by Cheng et al., which were measured on samples prepared from metakaolinite, blast furnace slag and a potassium silicate solution [48]. The geopolymer binder prepared from metakaolin (M1) had up to two times slower initial setting time than geopolymer binders prepared from M2 and M3 (calcined kaolinite claystones) at a temperature of 25 °C. The same phenomenon can be observed in the case of real setting times. Differences were probably caused by the above-mentioned morphology (different size, number and shape) of aluminosilicate particles after partial dissolution, which resulted in a subsequent faster geopolymerization process [21]. The setting time could also be influenced by chemical composition (higher content of impurities in calcined kaolinite claystones) of the raw materials. There were no significant differences between samples GB-M2 and GB-M3.

**Figure 4:**
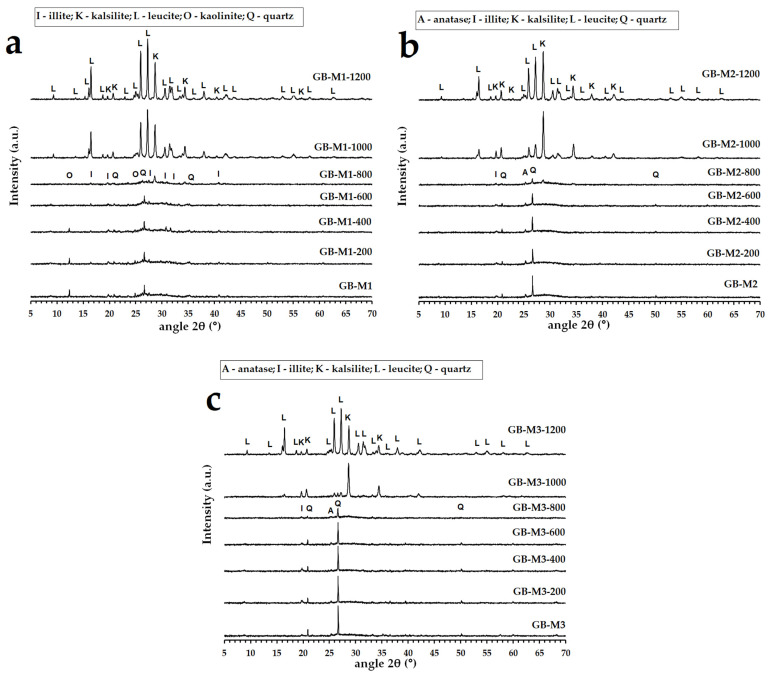
XRD patterns of the geopolymer binders with different type of aluminosilicate ((**a**)—GB-M1, (**b**)—GB-M2, (**c**)—GB-M3) at laboratory temperature and after exposure up to 1200 °C.

The XRD patterns of geopolymer binders tested after hardening and after exposure to temperatures elevated up to 1200 °C are reproduced in Figure 4. The diffractograms for all the geopolymer binders at laboratory temperature showed mainly amorphous phases. The presence of crystalline kaolinite (Al_2_Si_2_O_5_(OH)_4_) in the GB-M1 sample can be explained by the incomplete calcination of kaolin to metakaolin (amorphous metakaolinite). The X-ray diffraction patterns of GB-M1, GB-M2 and GB-M3 did exhibit the presence of some impurities, such as quartz (SiO_2_), anatase (TiO_2_) and illite (KAl_2_SiO_3_AlO_10_(OH)_2_). Impurities can be observed in raw aluminosilicate materials (see Figure 1). The phase composition changed after geopolymer binders were exposed to 1000 °C. Crystalline phases of kalsilite (KAlSiO_4_) and leucite (KAlSi_2_O_6_) were formed. Leucite and kalsilite are typical crystalline phases for potassium geopolymers exposed to high temperatures [49,50,51,52]. The effect of different types of aluminosilicates had no effect on the crystallization temperature or the formation of other phases. 

**Figure 5:**
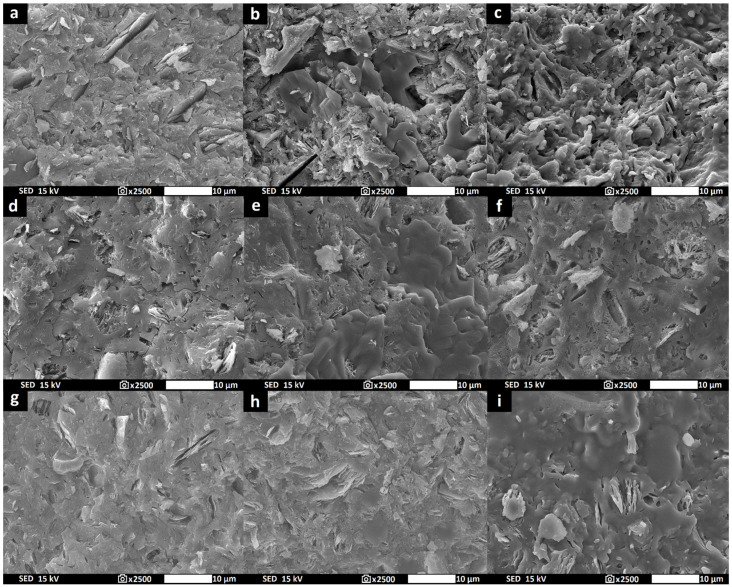
Micrographs of geopolymer binders with different type of aluminosilicate at laboratory temperature ((**a**)—GB-M1, (**d**)—GB-M2, (**g**)—GB-M3) after exposure to 800 °C ((**b**)—GB-M1, (**e**)—GB-M2, (**h**)—GB-M3) and 1000 °C ((**c**)—GB-M1, (**f**)—GB-M2, (**i**)—GB-M3).

Figure 5 shows the microstructure transformation of geopolymer binders at laboratory temperature and after heat treatment at 800 °C and 1000 °C observed with a scanning electron microscope. The unheated geopolymer binders hardened at laboratory temperature presented an amorphous, inhomogeneous and compact geopolymer matrix with undissolved metakaolinite plates. The geopolymer matrix did not contain a large number of visible pores. However, after being subjected to heat of 800 °C, the geopolymer matrices suffered microstructure transformations; a smooth structure could be seen, probably formed by crystallization of new phases of kalsilite and leucite (in accordance with the above XRD results). An inhomogeneous amorphous matrix with undissolved plates was still partially present. The microstructure of geopolymer binders after heat treatment at 1000 °C was manifested by the formation of a homogeneous and porous structure. This structure was created due to the crystallization of the geopolymer matrix. The crystallization of the geopolymer matrix after exposure to high temperature was also documented by previous works [52,53]. No significant difference was observed between individual samples with different types of aluminosilicates.

### 3.2. Geopolymer Composites’ Characteristics

**Figure 6:**
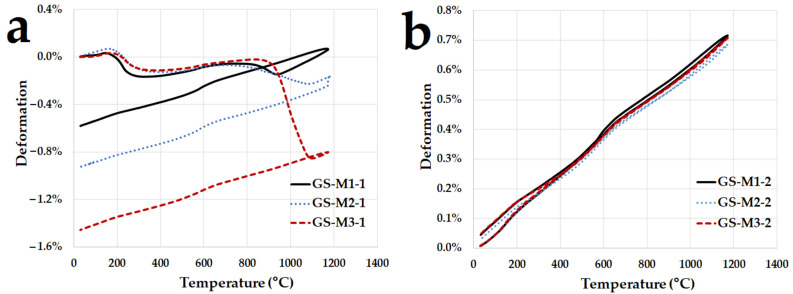
The dilatometric curves of first (**a**) and second (**b**) runs of geopolymer composites with different type of aluminosilicate up to 1200 °C (5 °C/min).

Figure 6a shows the curves of thermal dilatometry (first run) of the geopolymer composites with three different types of aluminosilicates measured from temperature 30 °C to 1200 °C at a heating speed of 5 °C/min. The dilatometric curves demonstrated that the effect of the aluminosilicate used was noticeable only from a temperature of 900 °C. As has been previously observed [54,55], the geopolymer shows slight shrinkage between approximately 100 and 300 °C, ascribed to the water loss from the geopolymer matrix. In the temperature range from 300 to 900 °C, a slight expansion was observed for all geopolymer composites due to the compensation of the shrinkage of the geopolymer matrix caused by the dehydroxylation and expansion of the filler. Interestingly, the shrinkage of the geopolymer samples at approximately 900 °C tends to accelerate, which was called viscous sintering [10,56]. The shrinkage was caused by the formation of new phases of kalsilite and leucite. Geopolymer composites experienced slight expansions up to the temperature limit of the experiment (1200 °C) from a temperature of 1100 °C (in the case of the GS-M1 sample, from 950 °C). The samples with calcined kaolinitic claystones (GS-M2 and GS-M3) showed a more significant shrinkage compared to the sample prepared from metakaolin (GS-M1). The geopolymer composites shrank linearly during cooling. The total shrinkage of the geopolymer composite samples during the first run was approximately 0.6% (GS-M1), 0.9% (GS-M2) and 1.5% (GS-M3) from their length after completing the first heating and cooling. A total shrinkage of around 1% of the metakaolin geopolymer composite filled with chamotte was also documented by Rovnaník et al. [7].

The curves of the second run of dilatometry of geopolymer composites are presented in Figure 6b. It can be seen from the dilatometric curves that the geopolymer composites expanded linearly, and both the heating and cooling curves of dilatometry in the second run followed the cooling curves in the first run. The linear coefficients of thermal expansion (α) of samples GS-M1, GS-M2 and GS-M3 were 6.11, 5.76 and 5.72 × 10^−6^/°C, while the values were measured in the temperature range of 200 to 1000 °C during the second run of heating. It is evident that the influence of the type of aluminosilicate does not have an important effect on thermal expansion during repeated heating.

**Figure 7:**
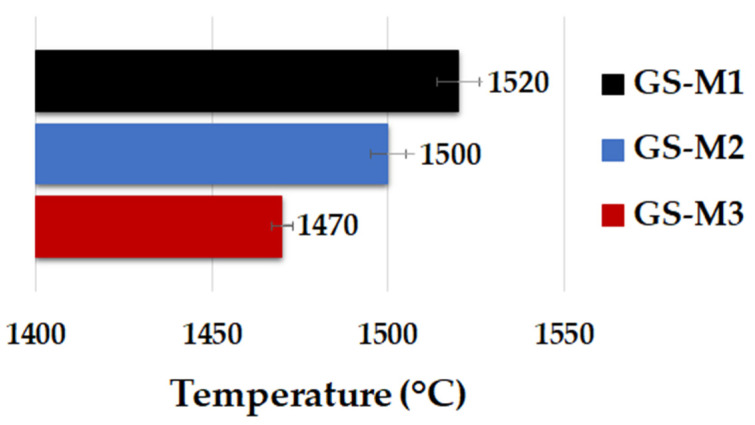
Pyrometric cone refractoriness of geopolymer composites with different types of aluminosilicates.

Figure 7 displays the results of heat microscopy of geopolymer composites. The heat microscopy (pyrometric cone refractoriness) of geopolymer composites was determined by the raw aluminosilicate material used. The value of pyrometric cone refractoriness was higher for GS-M1 than for the samples prepared from calcined kaolinitic claystones (GS-M2 and GS-M3). For GS-M1, the refractory temperature was set at 1520 °C, for GS-M2 at 1500 °C, and for GS-M3 at 1470 °C. The different refractoriness values could be due to impurities in the aluminosilicate raw materials. The prepared geopolymeric composites can be considered refractory materials (high-temperature applications) due to the high values of pyrometric cone refractoriness [57].

**Figure 8:**
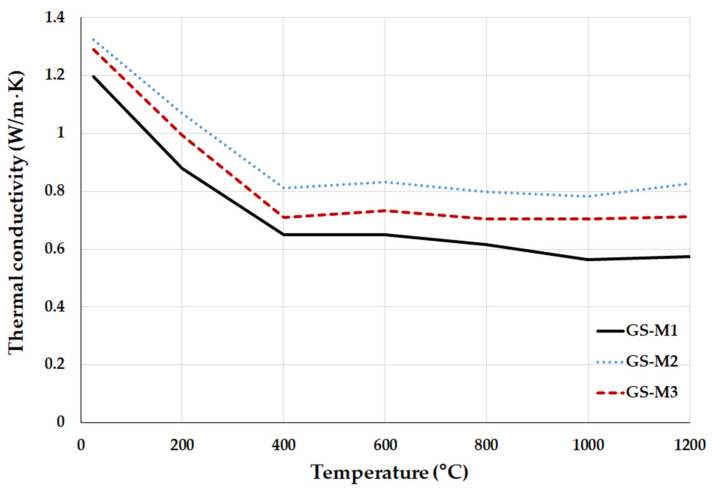
Thermal conductivity of geopolymer composites with different types of aluminosilicates at laboratory temperature and after exposure up to 1200 °C.

The thermal conductivity of geopolymer composites with different types of aluminosilicates as a function of the increasing exposure temperature is shown in Figure 8. Increasing the exposure temperature caused a significant decrease in thermal conductivity up to a temperature of 400 °C (from 1.2–1.3 to 0.8–0.65 W/m*K). The thermal conductivity of geopolymer composites decreases due to evaporation of free and chemically bound water from the pores of samples [58]. After the evaporation of water (from 400 °C), there was no notable decrease in the thermal conductivity of the examined samples. The thermal conductivity values obtained by other authors for the metakaolin-based geopolymer binders are 0.3–0.7 W/m*K [28,59]. The thermal conductivity of chamotte is around 1 W/m*K [60]. The results of thermal conductivity of geopolymer composites filled with chamotte were due to a combination of thermal conductivity of the geopolymer binder and filler. Geopolymer composites prepared from calcined kaolinitic claystones (GS-M2 and GS-M3) had a slightly higher thermal conductivity than the composite prepared from metakaolin (GS-M1). 

**Figure 9:**
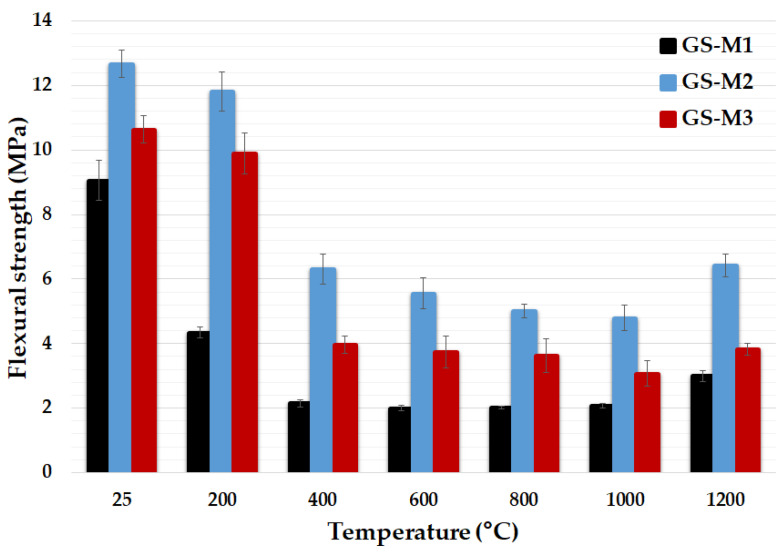
Flexural strength of the geopolymer composites with different types of aluminosilicates at laboratory temperature and after exposure up to 1200 °C.

Figure 9 provides the results of flexural strength of geopolymer composites with different types of aluminosilicates tested at laboratory temperature and after heat treatment up to 1200 °C. All the tested samples of geopolymer composites had a decrease in flexural strength with increasing temperature up to a temperature of 1000 °C. The residual flexural strengths of GS-M1, GS-M2 and GS-M3 were about 24%, 50% and 37% at 400 °C, and for the unheated samples, the flexural strengths were 9.1, 12.7 and 10.6 MPa, respectively. The dehydration of the tested samples, which caused the formation of cracks, was the main reason for the significant decrease in flexural strength between the laboratory temperature and 400 °C [27,58]. The flexural strength of all samples increased at a temperature of 1200 °C. The flexural strengths of GS-M1, GS-M2 and GS-M3 were about 45%, 34% and 25% higher than their flexural strengths as tested at 1000 °C (2.1, 4.8 and 3.1 MPa). The crystallization of new phases (leucite and kalsilite) or sintering of the matrix could be the main reasons for the increase in flexural strength. The effect of aluminosilicate on flexural strength is evident; samples prepared from calcined kaolinitic claystones had better flexural strengths compared to samples from calcined kaolin. The highest flexural strengths at all temperatures were achieved in sample GS-M2 (25 °C–12.7 MPa; 400 °C–6.3 MPa; 1200 °C–6.4 MPa), followed by sample GS-M3 (25 °C–10.6 MPa; 400 °C–3.96 MPa; 1200 °C–3.8 MPa) and GS-M1 (25 °C–9.0 MPa; 400 °C–2.15 MPa; 1200 °C–3.0 MPa). Amin et al. [61] observed similar results of flexural strength (around 9.0 MPa) for metakaolinite-based geopolymer. These findings are in agreement with the findings from previous studies, which reported that the flexural strength of metakaolinite-based geopolymer decreases with elevated temperature [20,62]. 

**Figure 10:**
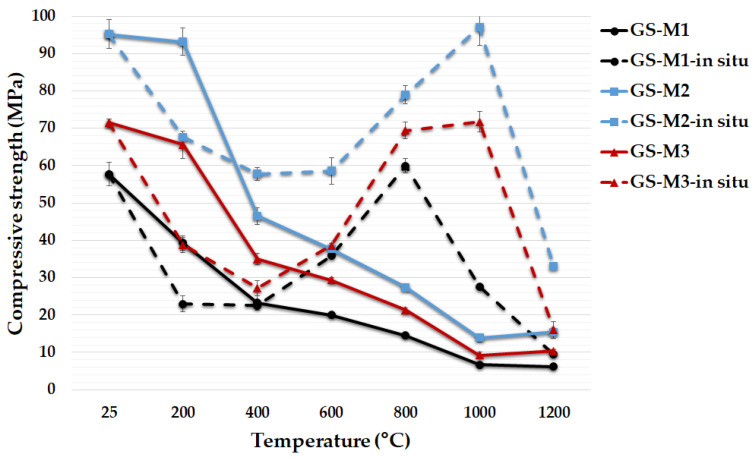
Compressive strength of the geopolymer composites with different types of aluminosilicates at laboratory temperature, after exposure up to 1200 °C and in situ temperature from 25 °C to 1200 °C.

Figure 10 shows the average values of compressive strength of three types of geopolymer composites with various aluminosilicates before and after exposure to elevated temperatures and in situ temperatures of 200 °C, 400 °C, 600 °C, 800 °C, 1000 °C and 1200 °C. A significant decrease in compressive strength of geopolymer composites was observed after exposure to elevated temperatures, as in the case of flexural strength. This finding was also observed by other authors [9,10,20,42,62]. A severe loss of compressive strength was observed especially in the temperature range of 200–400 °C for geopolymer composites. The residual compressive strengths of GS-M1, GS-M2 and GS-M3 were about 40%, 49% and 49% after exposure to 400 °C from the results of compressive strength (57.7 MPa, 95.2 MPa and 71.5 MPa, respectively) tested at laboratory temperature. Dehydration of the geopolymer matrix caused a severe loss of compressive strength [58]. Further loss in compressive strength was due to dehydroxylation of the geopolymer matrix [27]. An apparent influence of the type of aluminosilicate on the compressive strength can be seen in the temperature range of 25 °C to 400 °C. Samples prepared from M2 and M3 had significantly higher compressive strengths than samples prepared from M1, as in the case of flexural strength. There were no longer significant differences between the tested samples from a temperature of 400 °C, and with increasing temperature, the differences decreased. The highest compressive strength at laboratory temperature was reached in sample GS-M2 (95.2 MPa), followed by GS-M3 (71.5 MPa) and GS-M1 (57.7 MPa). The investigated samples of geopolymer composites had comparable compressive strength results, approximately 11 MPa, after exposure to a high temperature of 1200 °C. Amin et al. [61] conducted research on the behavior of high-strength metakaolinite-based geopolymer composite under high temperature. According to the results of this research, the values of compressive strength at elevated temperatures (55 MPa at 400 °C, 45 MPa at 600 °C) were a little higher than the results obtained in this study. On the contrary, in the research of Aygörmez et al. [62], the results of compressive strength (25 MPa at 600 °C, 9 MPa at 900 °C) were lower. Differences in compressive strength values were due to different chemical composition (Si/Al) or using a different alkaline activator (sodium instead of potassium).

The described experiment was also aimed at investigating the compressive strength of geopolymer composites at high temperature tested in situ. Figure 10 shows that the dependences of the compressive strength on temperature, tested in situ, did not match the above-described dependences of the sample tested after heat exposure and cooling. The previously described dehydration also caused a decrease in the compressive strength of the investigated geopolymer composites in situ, up to a temperature of 400 °C. The change occurred from a temperature of 600 °C, when the compressive strength of all examined samples increased up to a temperature of 1000 °C (800 °C, in the case of GS-M1). The increase in compressive strength after exposure to temperatures higher than 400 °C can be attributed to the absence of a shrinkage phase during cooling or to increasing the plasticity of the tested samples [46]. There was a severe loss of compressive strength after exposure to 1200 °C (1000 °C), which can probably be attributed to the above-mentioned crystallization of new phases (leucite and kalsilite) or sintering. The same phenomenon was observed in the testing of in situ, high-temperature compressive strength, as in the case of compressive or flexural strength; samples GS-M2 (97.1 MPa at 1000 °C) and GS-M3 (71.9 at 1000 °C) had higher values of compressive strength than sample GS-M1 (29.1 MPa at 1000 °C). Higher values of mechanical strength can probably be attributed to particle morphology of aluminosilicate particles, which resulted in a higher solubility of calcined kaolinitic claystones and a subsequent faster geopolymerization process (calcined kaolinitic claystones had earlier initial setting time).

**Figure 11:**
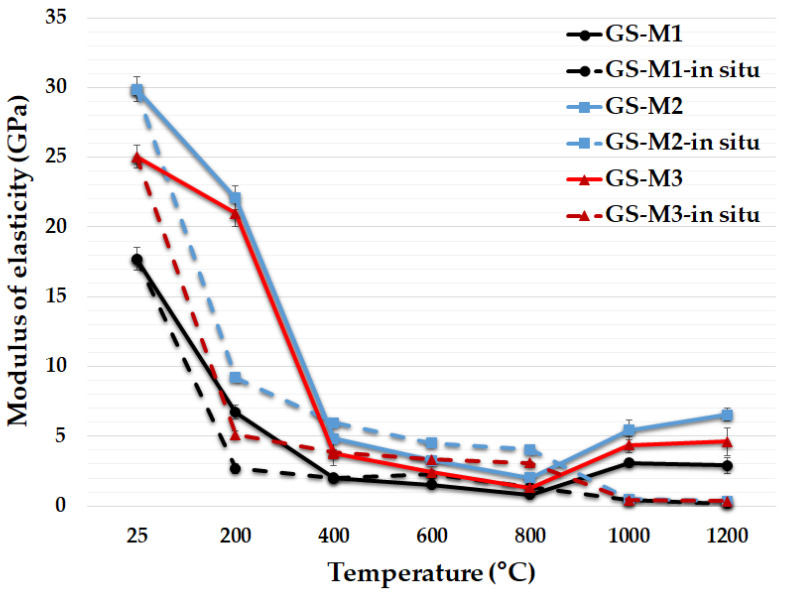
Modulus of elasticity of the geopolymer composites with different types of aluminosilicates at laboratory temperature, after exposure up to 1200 °C and in situ temperature from 25 °C to 1200 °C.

The effect of the three types of aluminosilicates on the results of modulus of elasticity of the prepared geopolymer composites, before and after exposure to elevated temperatures and in situ temperatures in the range of 200 °C to 1200 °C, can be seen in Figure 11. A substantial loss of modulus of elasticity with higher temperature, especially in the temperature range of 25 °C to 400 °C, and higher values of modulus of elasticity for samples prepared from calcined kaolinitic claystones (M2 and M3) compared to metakaolin (M1) were observed. The trends of the curves correspond with the results of compressive strength. Geopolymer composites GS-M1, GS-M2 and GS-M3 had a modulus of elasticity of 17.7, 29.9 and 25.1 GPa at laboratory temperature and 2.9, 6.5 and 4.6 GPa after exposure to 1200 °C. Amin et al. [61] produced a metakaolinite-based geopolymer composite with a modulus of elasticity around 30 GPa at laboratory temperature.

Figure 11 also displays the modulus of elasticity of geopolymer composites tested in situ in the temperature range from 25 to 1200 °C. The modulus of elasticity tested in situ decreased with increasing temperature, analogously to the samples tested after exposure to high temperature and cooling. The values of the modulus of elasticity were almost zero after reaching the temperature of 1000 °C. The samples had a more plastic behavior; therefore, their modulus of elasticity was almost zero, and their compressive strength tested in situ was high. The plastic behavior was caused by the already mentioned crystalline changes.

## 4. Conclusions

In this research, the effect of different types of aluminosilicates on the thermo-mechanical properties of metakaolinite-based geopolymer binders and composites (filled with chamotte) was examined. The findings from this study resulted in the following conclusions:

- The dynamic viscosities of the fresh geopolymer binder based on metakaolin (1090 mPa·s) were significantly higher than the viscosities of the binders based on calcined kaolinitic claystones (787 and 588 mPa·s). The initial setting time of geopolymer binders based on calcined kaolinitic claystones (250 and 267 min) was two times faster compared to the geopolymer binder based on metakolin (516 min).
- No significant differences between the examined geopolymer binders were observed in XRD diffractograms at laboratory temperature and after exposure to elevated temperatures.
- Geopolymer composites based on calcined kaolinitic claystones showed slightly higher shrinkage during first heating (0.9% and 1.5%) and lightly lower refractoriness (1500 and 1470 °C) than geopolymer composites based on metakaolin (0.6% and 1520 °C).
- The results of mechanical properties showed that geopolymer composites prepared from calcined kaolinitic claystones had better mechanical properties (compressive strength 95.2 and 71.5 MPa) than the geopolymer prepared from metakaolin (compressive strength 57.7 MPa), including mechanical properties after thermal exposure and in situ temperature from 25 °C to 1200 °C.

The results of these experiments clearly indicate that geopolymers based on calcined kaolinitic claystones are promising materials. Their advantages compared to geopolymers based on metakaolin are their lower price, better mechanical properties at laboratory temperature and even after thermal exposure and also a significantly lower viscosity of geopolymer binders. All the materials examined withstand temperatures up to 1450 °C without a load and can be considered as alternative refractory materials.

## Figures and Tables

**Table 1:** Chemical composition (wt. %) of raw materials.

Material	Material Composition (%)													
	^a^ LOI	H_2_O	SiO_2_	Al_2_O_3_	Fe_2_O_3_	CaO	MgO	Na_2_O	K_2_O	TiO_2_	P_2_O_5_	ZrO_2_	SrO	Cr_2_O_3_
M1	2.63	-	52.70	40.10	0.75	0.17	0.32	0.06	2.24	0.76	0.08	-	0.01	0.01
M2	1.59	-	52.00	42.30	0.93	0.16	0.139	-	0.82	1.71	0.07	0.03	0.01	0.03
M3	1.46	-	52.70	38.40	3.83	0.29	0.32	-	1.38	1.29	0.17	0.01	0.03	-
Potassium silicate	-	62.2	25.2	0.04	0.75	-	-	0.25	12.4	-	-	-	-	-
Chamotte	0.06	-	53.8	41.0	1.53	0.19	0.15	0.05	0.91	1.98	0.09	0.04	-	0.03

^a^ LOI = Loss on ignition.

**Table 2:** Physical properties of raw materials.

Material	Specific Gravity	Bulk Density	Particle Size		Specific Surface Area (BET)
	(kg/m^3^)	(kg/m^3^)	d_50_ (µm)	d_90_ (µm)	(m^2^/g)
M1	2626	350	3.88	10.36	12.6
M2	2641	536	5.90	16.88	13.3
M3	2659	479	5.35	16.30	16.6
Chamotte	2687	1524	-	-	1.9

**Table 3:** The initial, final and real setting time of geopolymer binders produced with different aluminosilicates at 25 °C and 60 °C.

Measurement Conditions	Sample	IST	FST	RST
		(min)		
25 °C, 95% humidity	GB-M1	516	639	123
	GB-M2	250	299	49
	GB-M3	267	322	55
60 °C	GB-M1	62	73	11
	GB-M2	44	48	4
	GB-M3	45	53	8

IST—initial setting time; FST—final setting time; RST = FST − IST—real setting time.

## Data Availability

The data presented in this study are available on request from the corresponding author.
